# Evaluation of Elnady preserved tissues as a teaching aid for undergraduate animal science courses

**DOI:** 10.1093/tas/txae077

**Published:** 2024-05-07

**Authors:** Jay A Daniel, Isabella Kukor

**Affiliations:** Department of Animal Science, Berry College, Mount Berry, GA 30149, USA; Department of Animal Science, Berry College, Mount Berry, GA 30149, USA

**Keywords:** tissue preservation, undergraduate

## Abstract

The use of tissue specimens for undergraduate instruction is a very valuable tool. However, fresh tissue specimens are not always available and many common preservation techniques can result in discoloration, offensive odors, and/or dangerous chemical residues. The Elnady Technique was developed as a means to produce tissue specimens that “are realistic, durable, have no offensive odor, and are dry, soft and flexible” (Elnady, F.A. 2016 The Elnady Technique: An innovative, new method for tissue preservation. *Altex.* 33:237-242. doi:10.14573/altex.1511091). Briefly for soft tissue, specimens were preserved by fixing in formalin. The tissue specimen was then dehydrated with a series of acetone baths. Once the tissue was fully dehydrated, the specimen was impregnated in glycerin. Excess glycerin was then removed by draining followed by immersion in cornstarch. Cornstarch residue was removed with a soft brush, and the specimen was stored in a plastic bag. Multiple specimens (including the female reproductive tract of the cat, goat, horse, and sow; digestive tract of cat, chicken, and dog; 1-day-old lamb stomach; goat rumen, reticulum, omasum, and abomasum; and sheep heart and kidney) have been preserved and used in various animal science course laboratories (126 laboratory sections and 1,696 students at Berry College). Some of the specimens have been in use for seven years and are still in usable condition. Anonymously surveyed Berry College Animal Science Faculty strongly agreed or agreed that Elnady preserved tissues are a useful teaching aid (*n* = 5). The Elnady Technique has proven to be a useful means of preserving tissue samples used in undergraduate animal science courses.

## Teaser

A new tissue preservation technique was used to produce realistic, durable tissue samples for undergraduate Animal Science courses.

## Introduction

Historically at Berry College, fresh and formalin-preserved tissues have been used to aid in the instruction of a number of courses (for example: Introduction to Animal Science, Anatomy and Physiology, Reproductive Physiology, and Principles of Nutrition) for undergraduate animal science students. Fresh tissue samples are not always available, resulting in the use of preserved tissue samples. Tissue preservation (or fixation) provides the maintenance of proteins, carbohydrates, and overall structural relationships to provide a realistic model without tissue decomposition (Thavarajah et al., 2012). Formalin, which is widely used to preserve tissue specimens (Thavarajah et al., 2012), can lead to discoloration, hardening of tissues, and a harsh chemical odor. Further, formalin is a skin and eye irritant and a confirmed carcinogen (National Center for Biotechnology Information). Plastination for tissue preservation is an effective tool for teaching anatomy which reduces the exposure of staff and students to potentially harmful chemicals (Latorre et al., 2007). However, plastination techniques require specialized equipment and chemicals. The Elnady Technique is a cost-effective, safe, and realistic alternative approach developed by Dr. Fawzy Elnady at the University of Cairo to preserve tissues for educational and research purposes (Elnady, 2016). The purpose of this report is to evaluate the use of the Elnady Technique to preserve specimens in undergraduate animal science courses.

## Materials and Methods

### Summary of Elandy Technique of Tissue Preservation

Tissue samples for preservation were obtained either from a local abattoir or during a necropsy of natural mortalities. Tissues were preserved using the Elnady Technique which utilizes fixation in formalin solution, followed by dehydration with acetone, impregnation with glycerin, and curing with cornstarch (Elnady, 2016). Hollow specimens were filled with polyester fiber fill. Newly preserved specimens using Elnady Technique were placed in a sealable plastic bag for storage purposes. If the specimen developed a greasy or moist feel, it was dried with a paper towel (Elnady, 2016). Multiple tissue specimens based on teaching needs and availability have been preserved using the Elnady Technique at Berry College and placed in a common area available for faculty to use as appropriate for the teaching objectives. The number of course laboratory sections and students utilizing the tissue specimens were calculated using historic enrollment data for Introduction to Animal Science, Anatomy and Physiology, Reproductive Physiology, and Principles of Nutrition laboratories since the Elnady Technique preserved tissue were produced.

### Faculty Survey

Survey procedures were approved by the Berry College Institutional Review Board for Human Subjects Research. Berry College Animal Science Faculty excluding the author (JD; *n* = 8) were emailed the invitation (Appendix 1) to participate in a survey regarding tissue specimens preserved using the Elnady Technique. Faculty members who chose to participate were asked to complete a survey (Appendix 2), print the completed survey, and place the survey in a drop box located in a common room in the Berry College Animal Science Building. Surveys were collected and data was summarized using MS Excel.

## Results and Discussion

**Figure 1.**
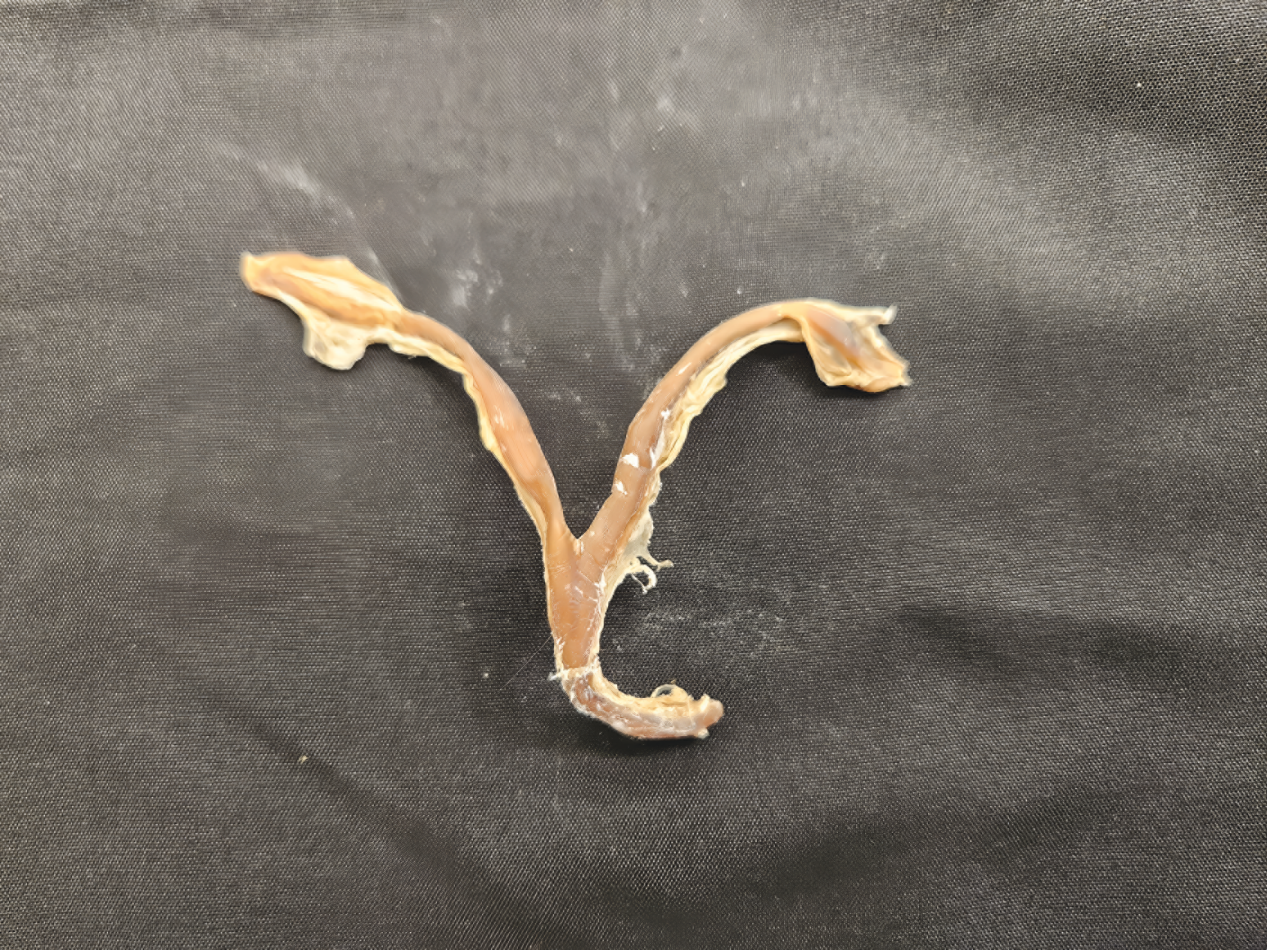
Female reproductive tract from a cat preserved using the Elnady Technique (Elnady, 2016) in 2017.

**Figure 2.**
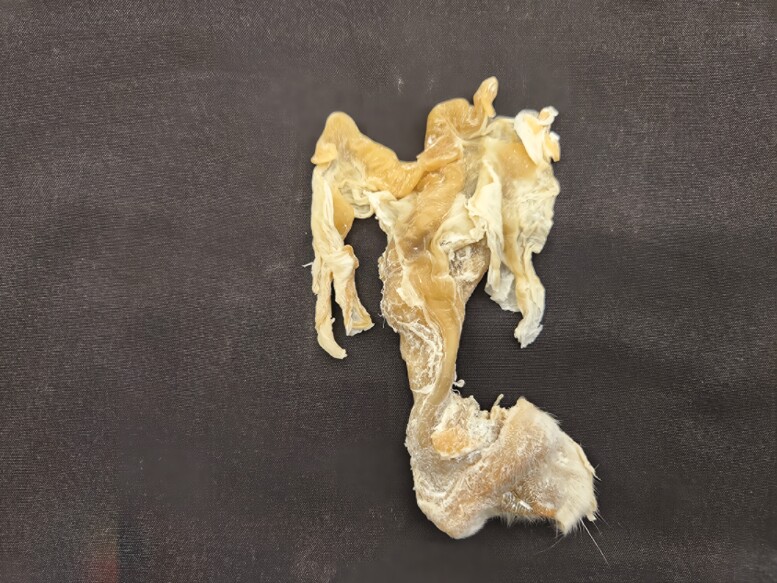
Female reproductive tract from a goat preserved using the Elnady Technique (Elnady, 2016) in 2017.

**Figure 3.**
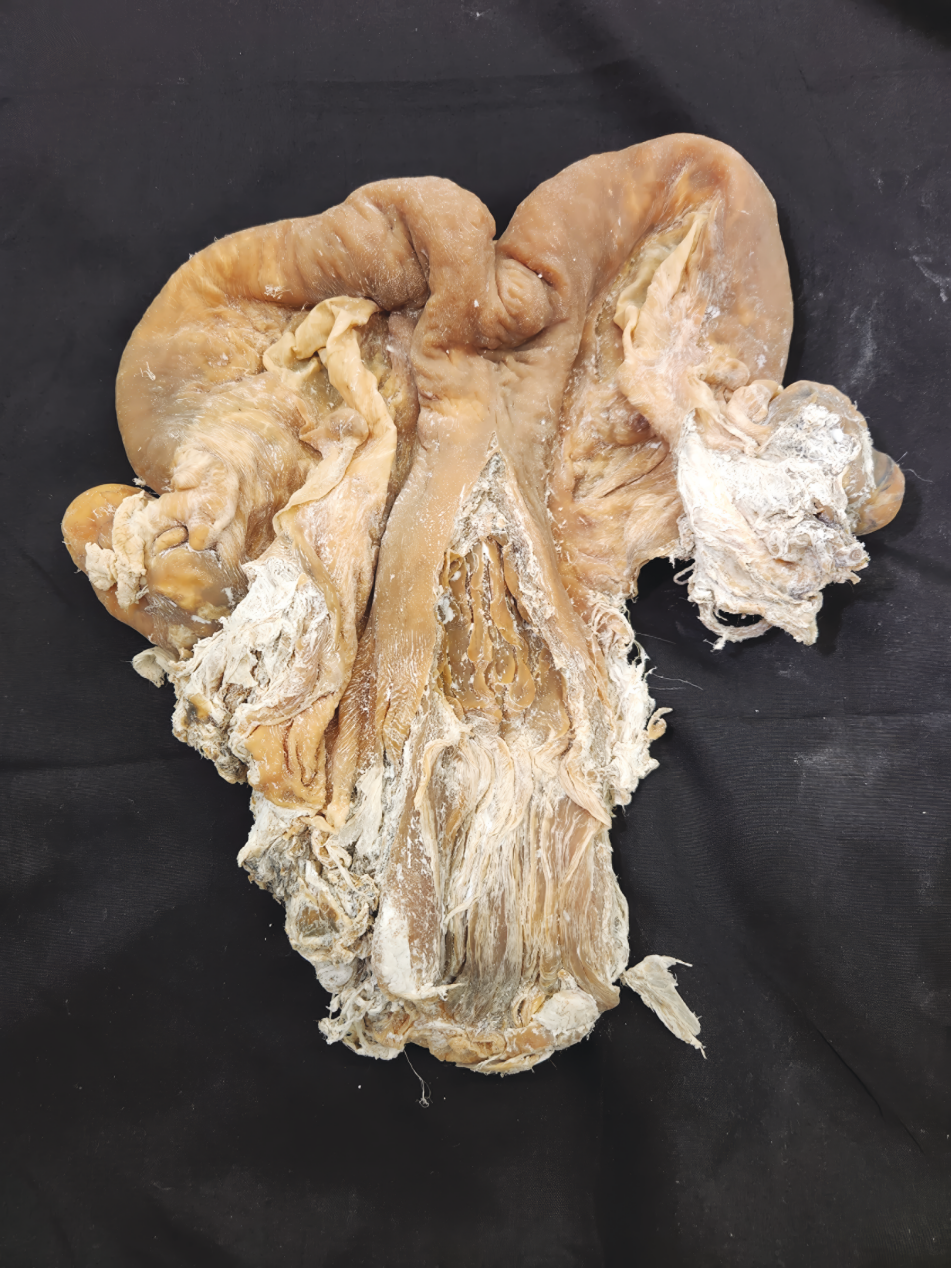
Female reproductive tract from a horse preserved using the Elnady Technique (Elnady, 2016) in 2019.

**Figure 4.**
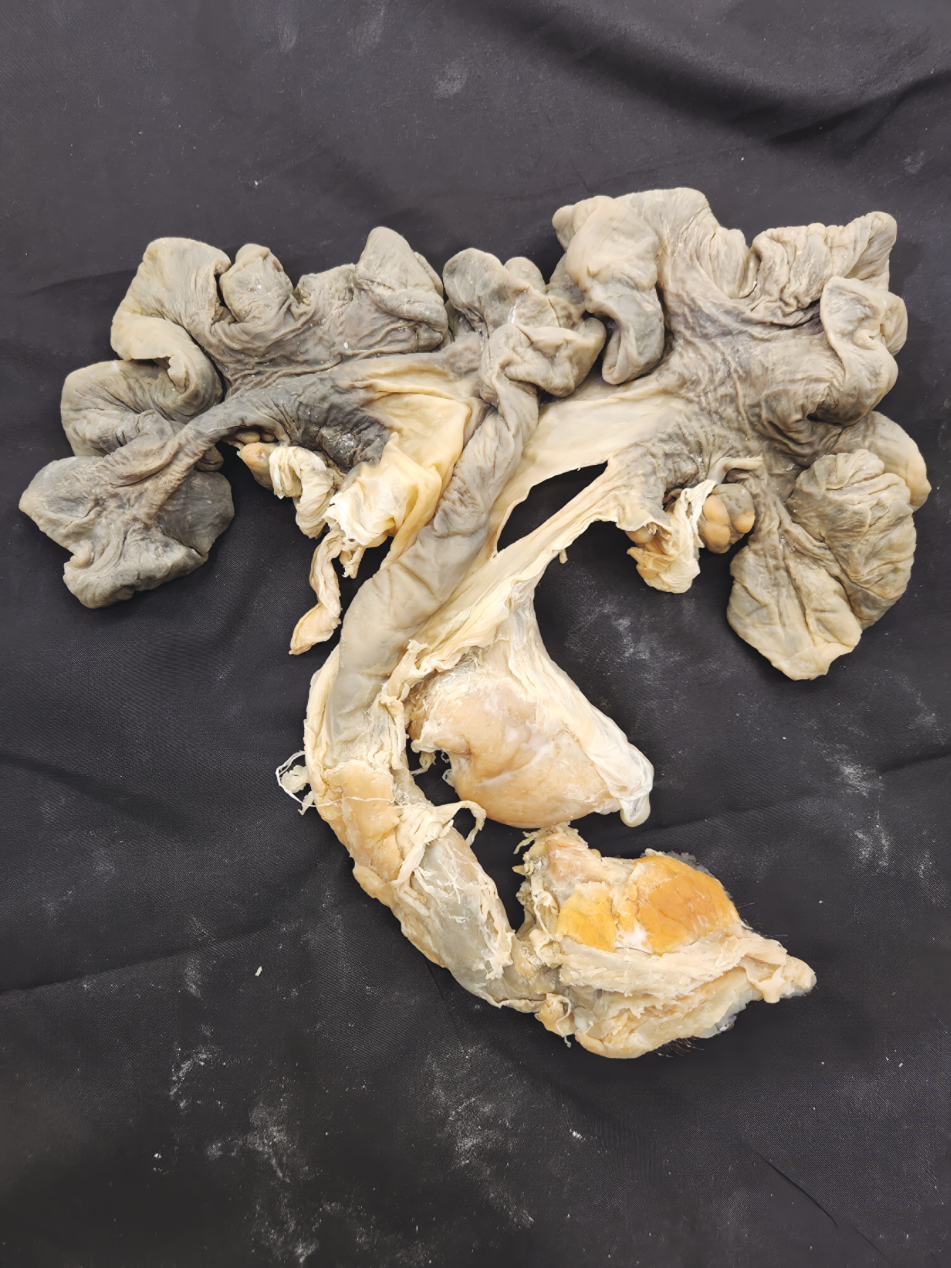
Female reproductive tract from a pig preserved using the Elnady Technique (Elnady, 2016) in 2017.

**Figure 5.**
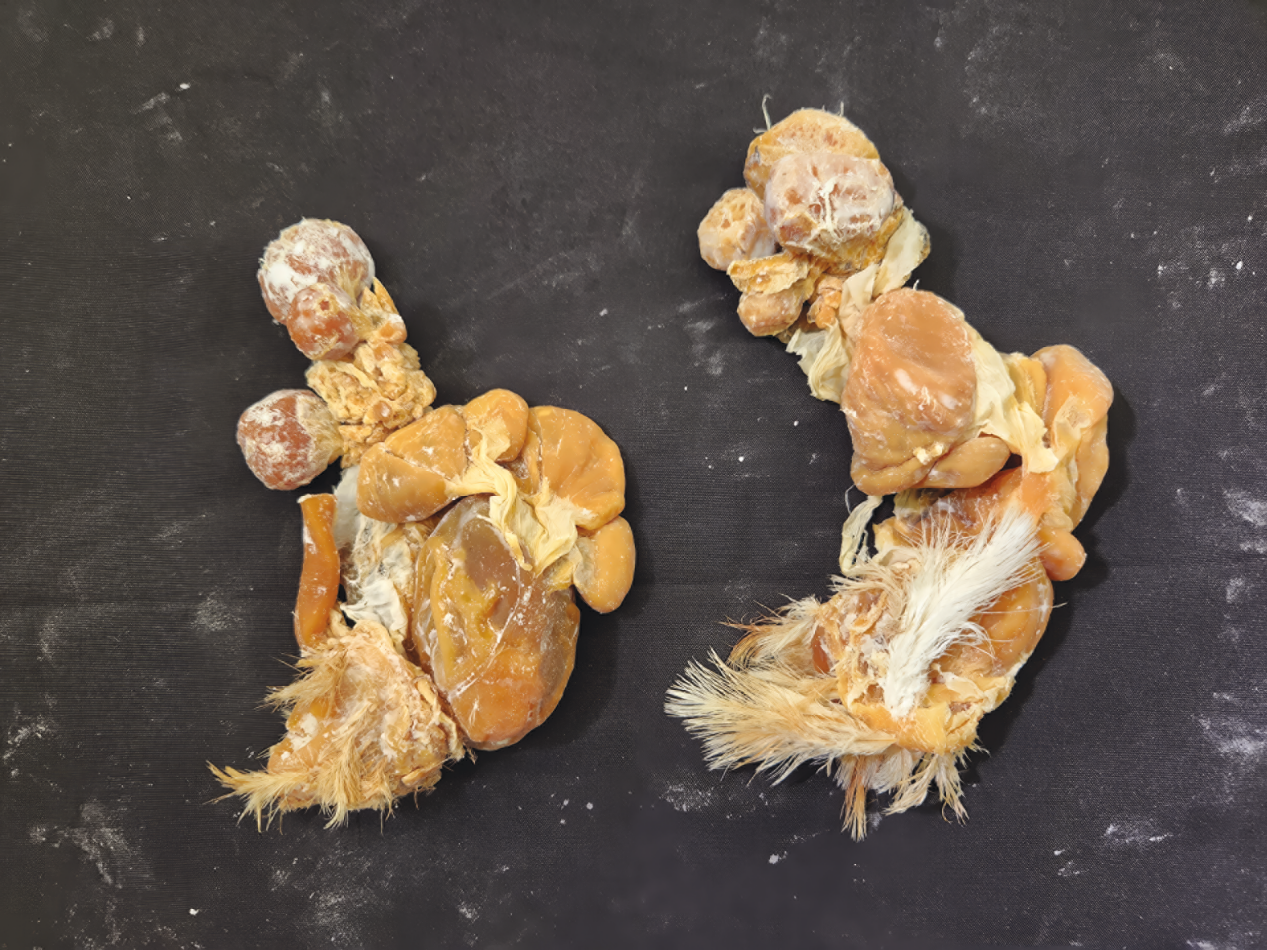
Female reproductive tract from two chickens preserved using the Elnady Technique (Elnady, 2016) in 2021.

**Figure 6.**
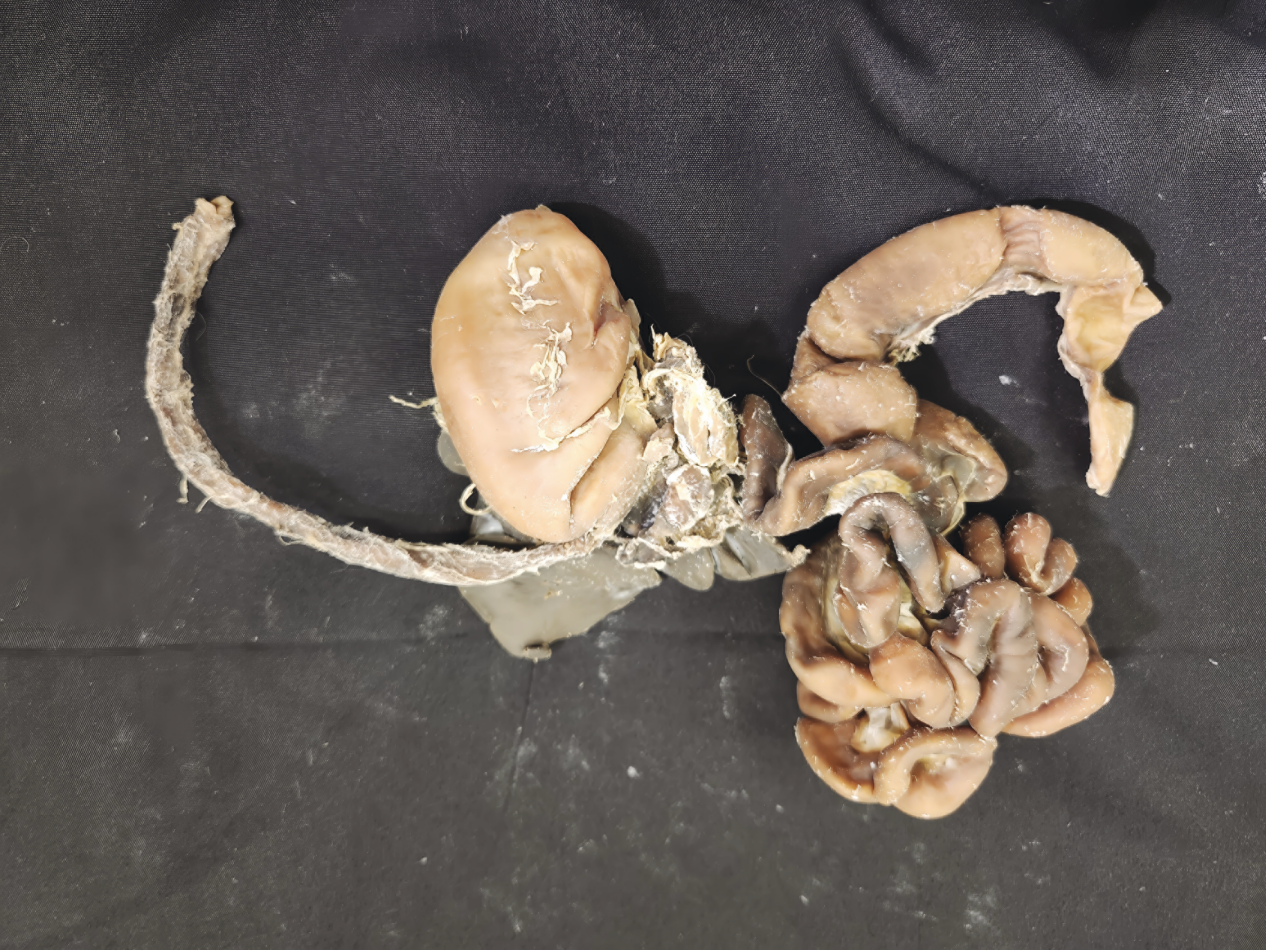
Digestive tract from a cat preserved using the Elnady Technique (Elnady, 2016) in 2021.

**Figure 7.**
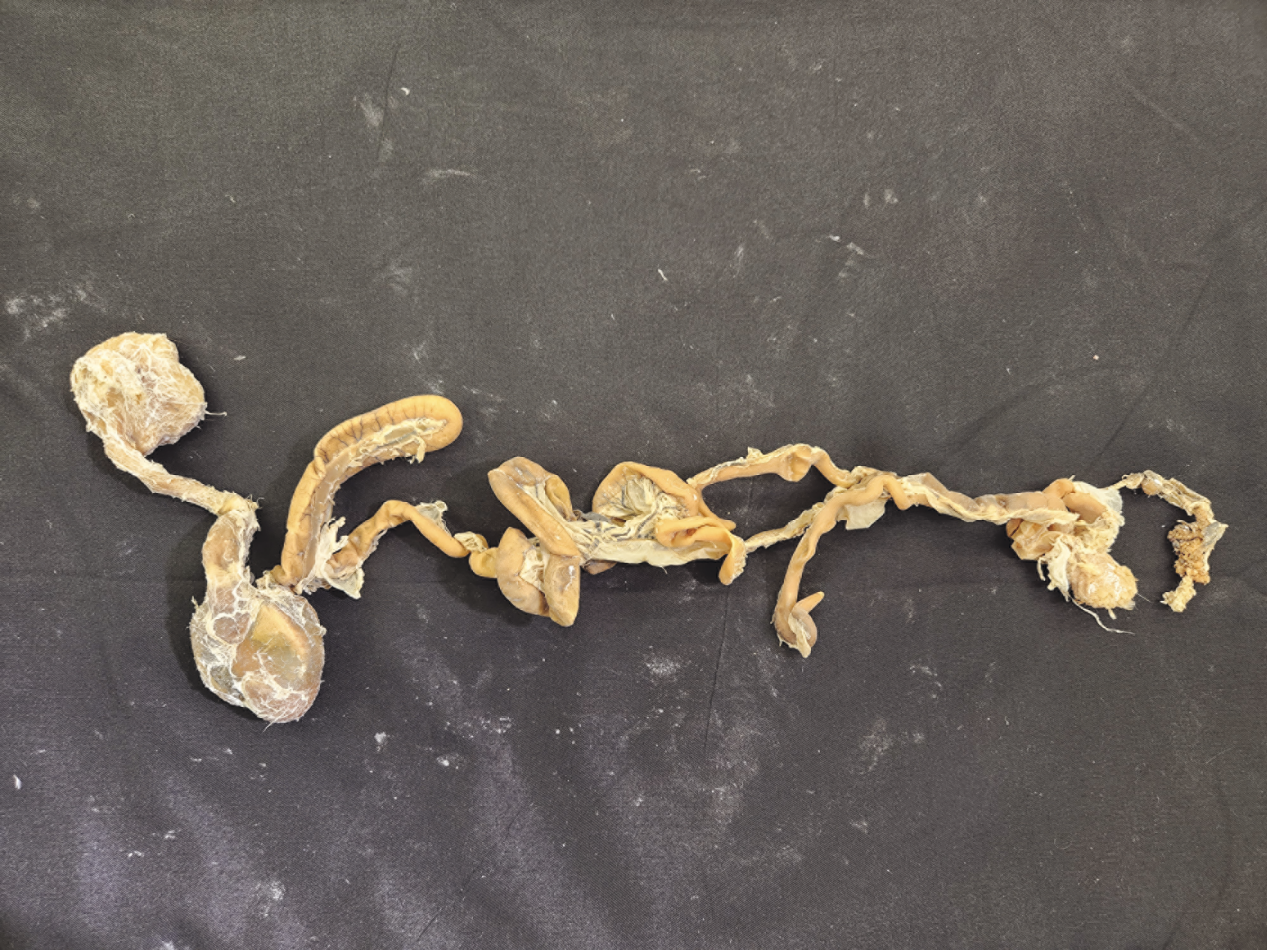
Digestive tract from a chicken preserved using the Elnady Technique (Elnady, 2016) in 2018.

**Figure 8.**
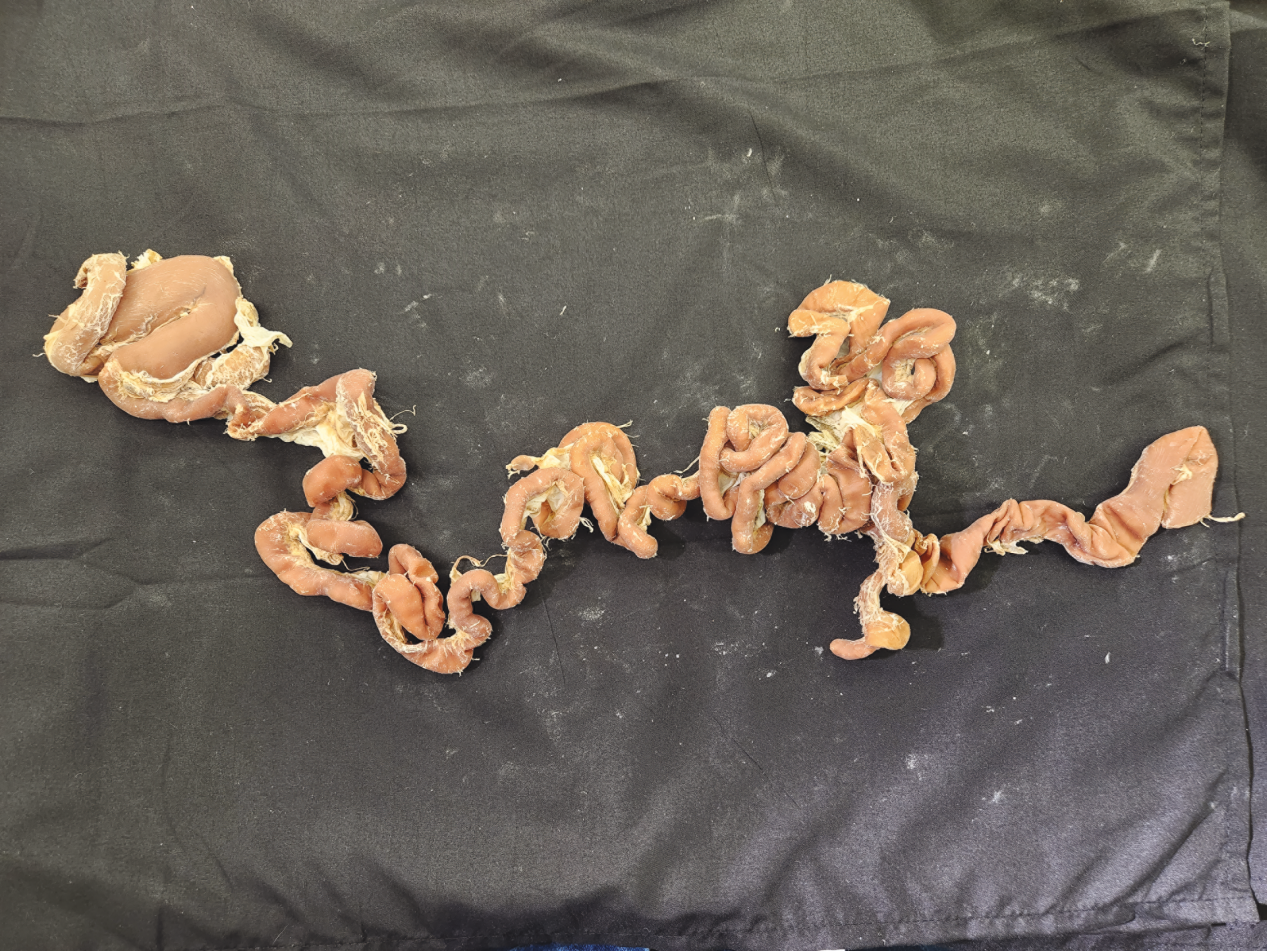
Digestive tract from a dog preserved using the Elnady Technique (Elnady, 2016) in 2021.

**Figure 9.**
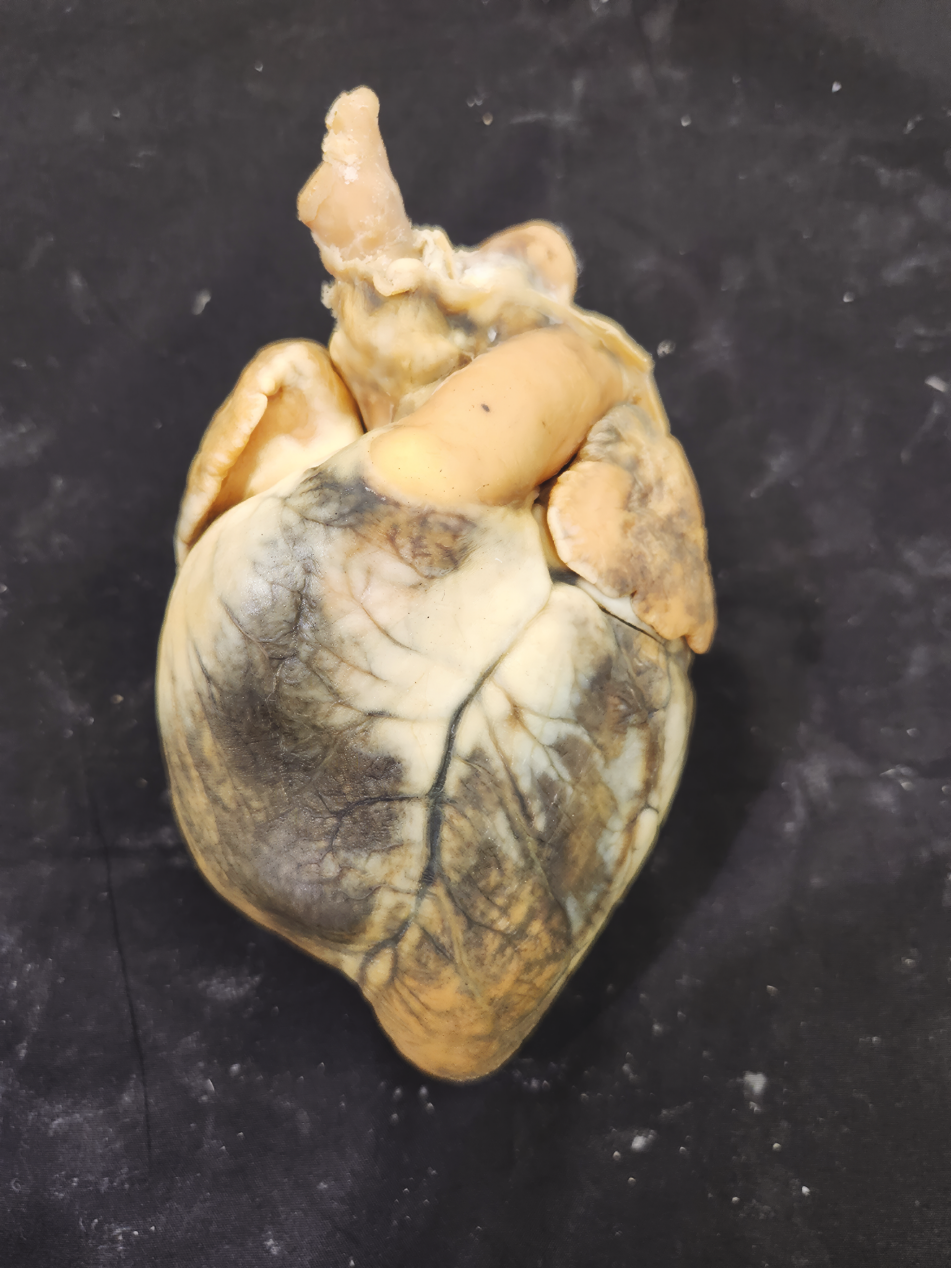
Heart of a sheep was preserved using the Elnady Technique (Elnady, 2016) in 2018.

**Figure 10.**
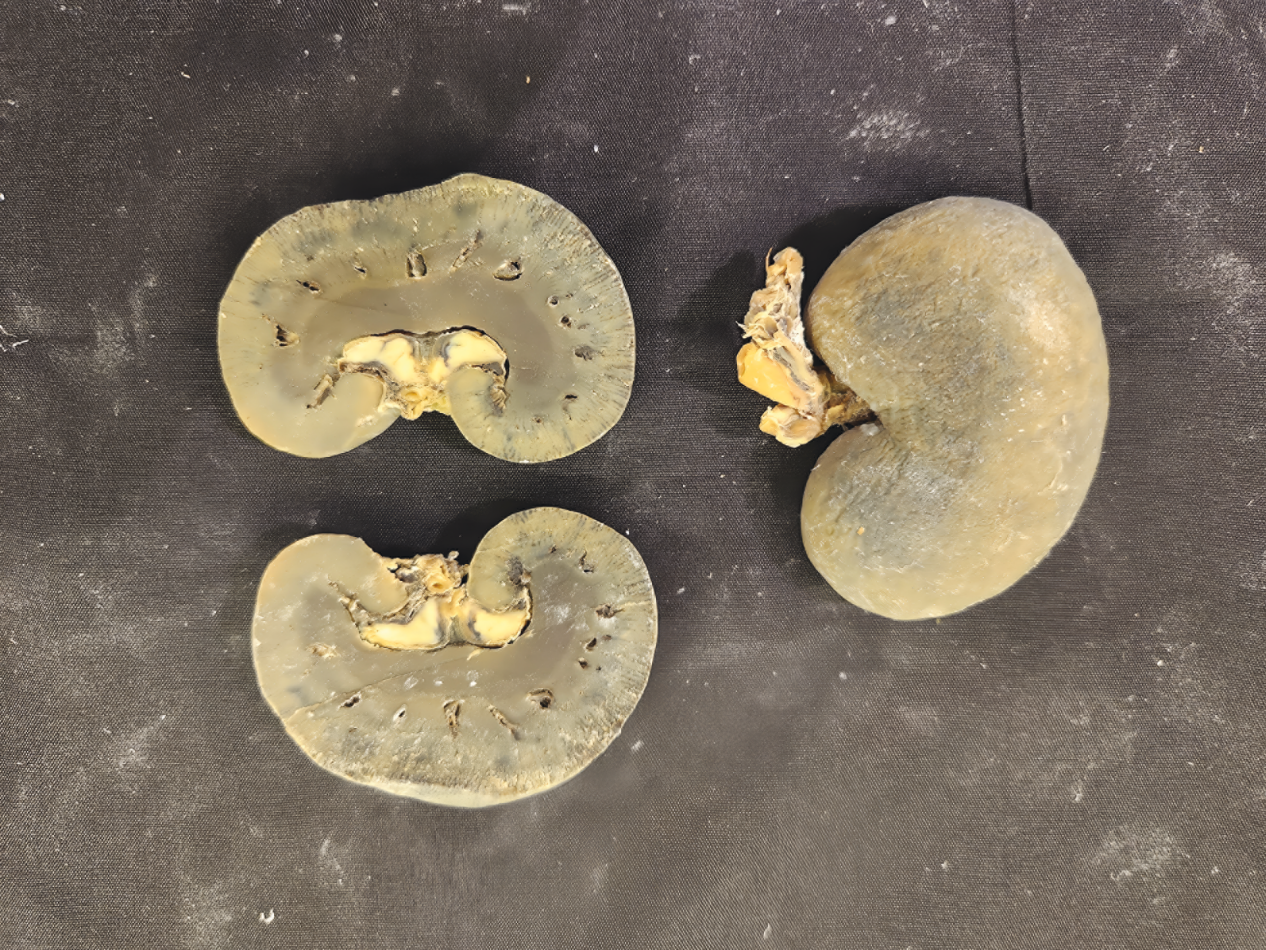
Kidneys from sheep were preserved using the Elnady Technique (Elnady, 2016) in 2018.

**Figure 11.**
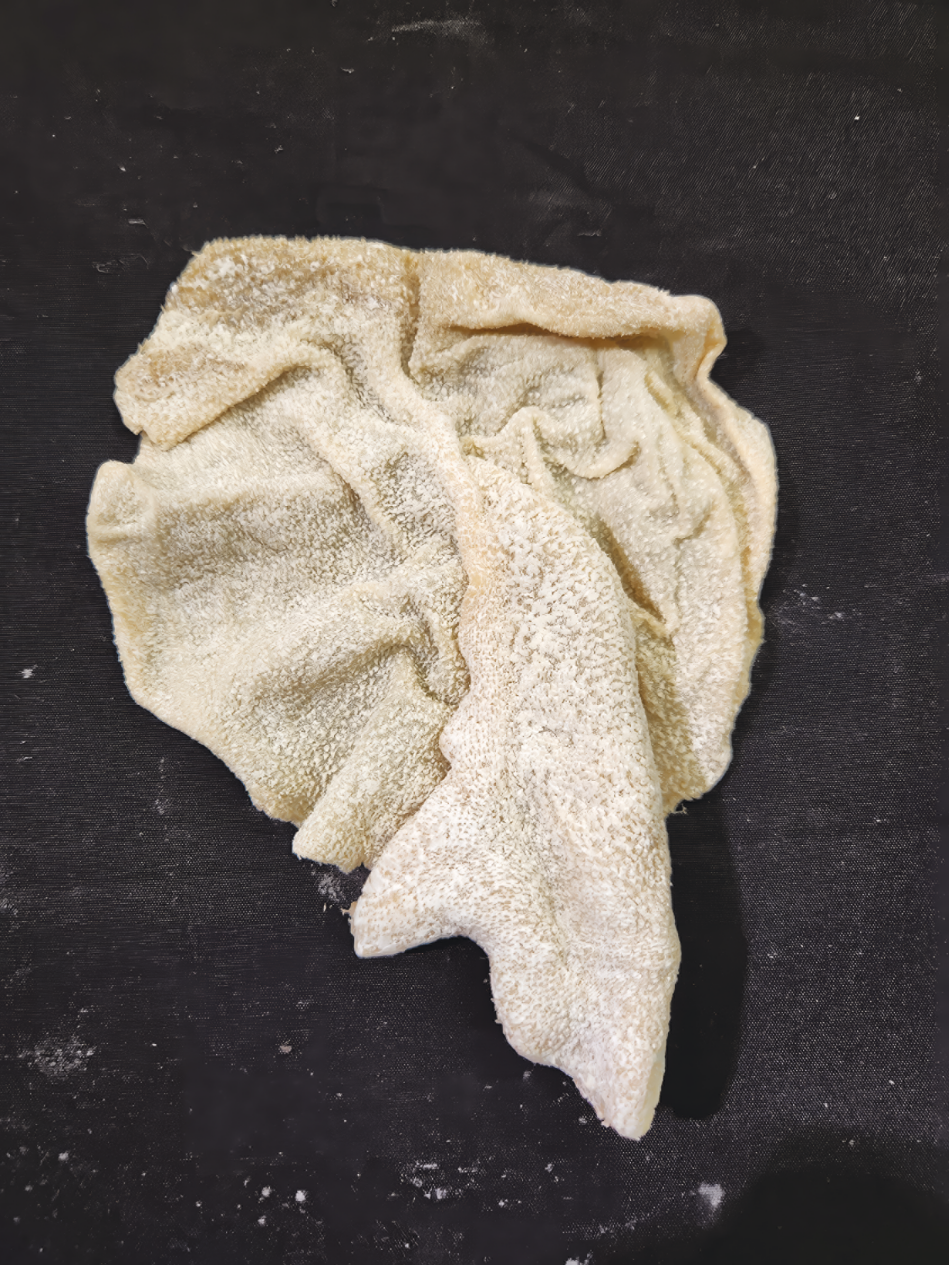
Rumen tissue from a goat was preserved using the Elnady Technique (Elnady, 2016) in 2017.

**Figure 12.**
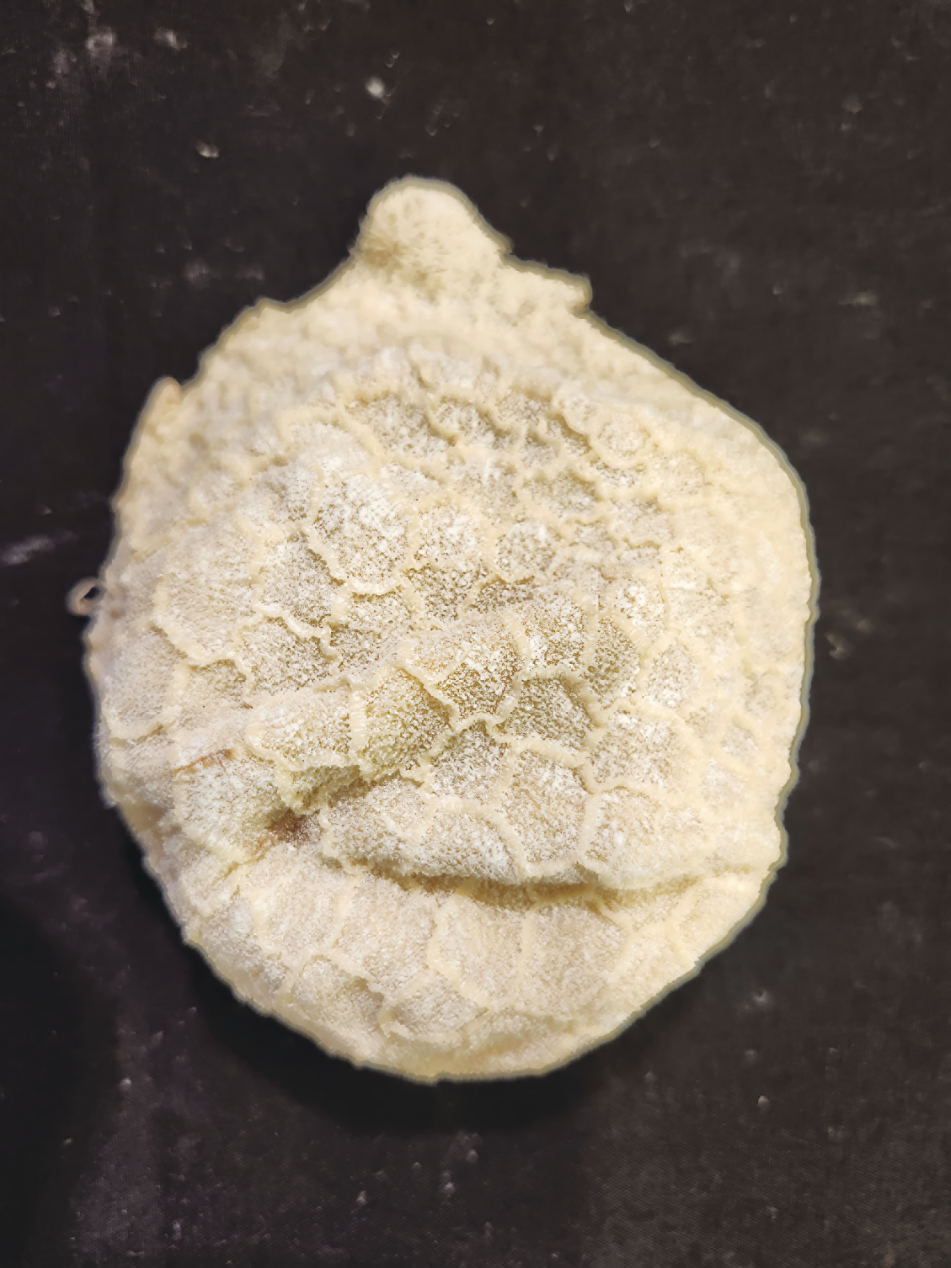
Reticulum tissue from a goat was preserved using the Elnady Technique (Elnady, 2016) in 2017.

**Figure 13.**
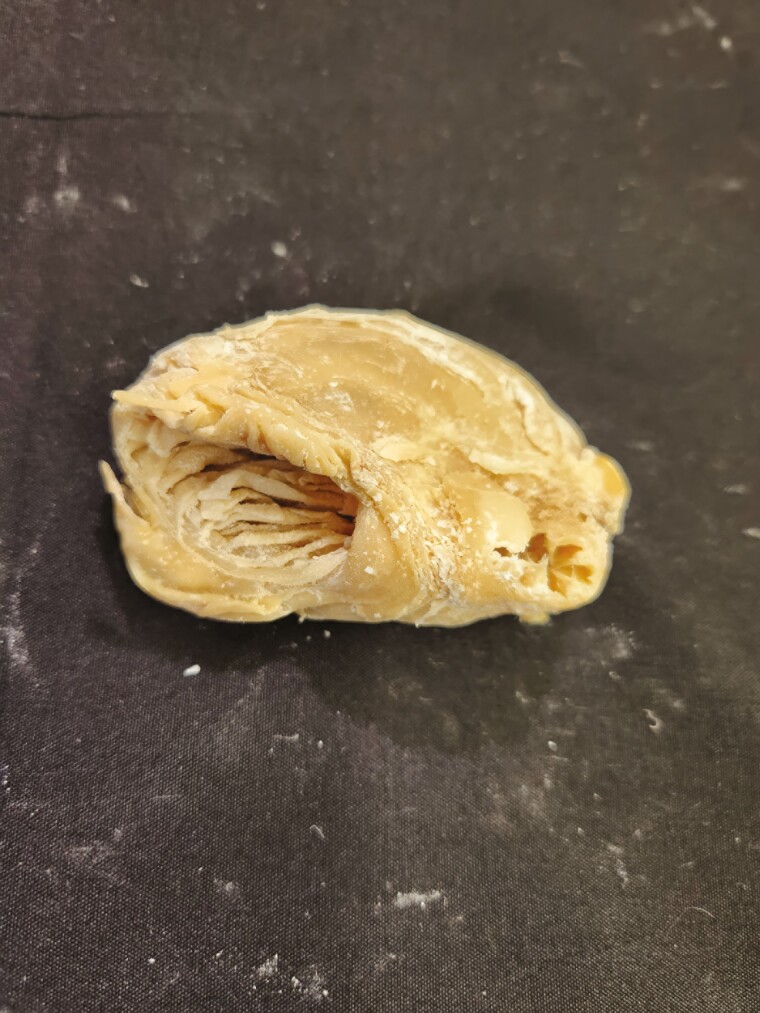
Omasum tissue from a goat was preserved using the Elnady Technique (Elnady, 2016) in 2017.

**Figure 14.**
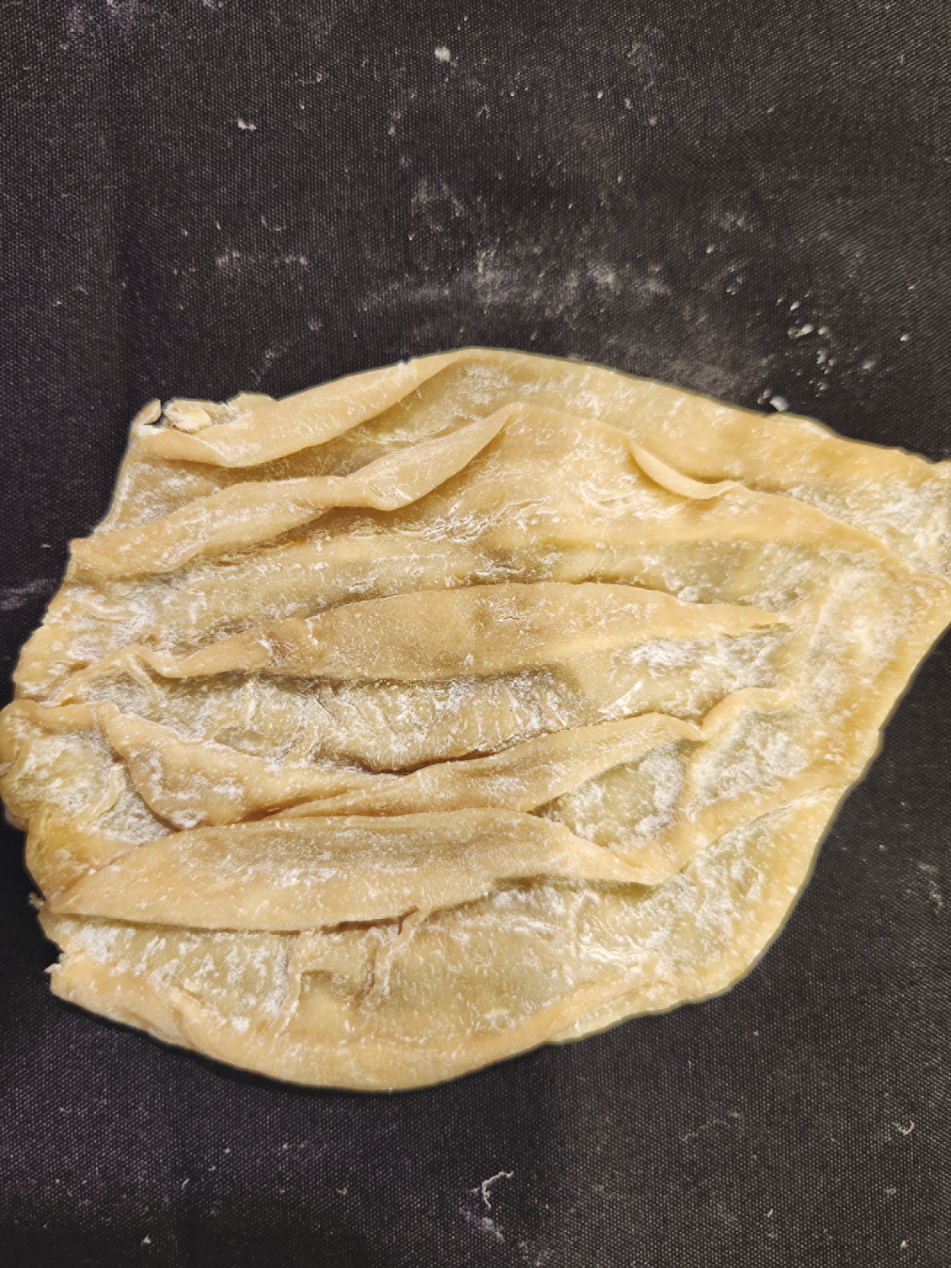
Abomasum tissue from a goat was preserved using the Elnady Technique (Elnady, 2016) in 2017.

**Figure 15.**
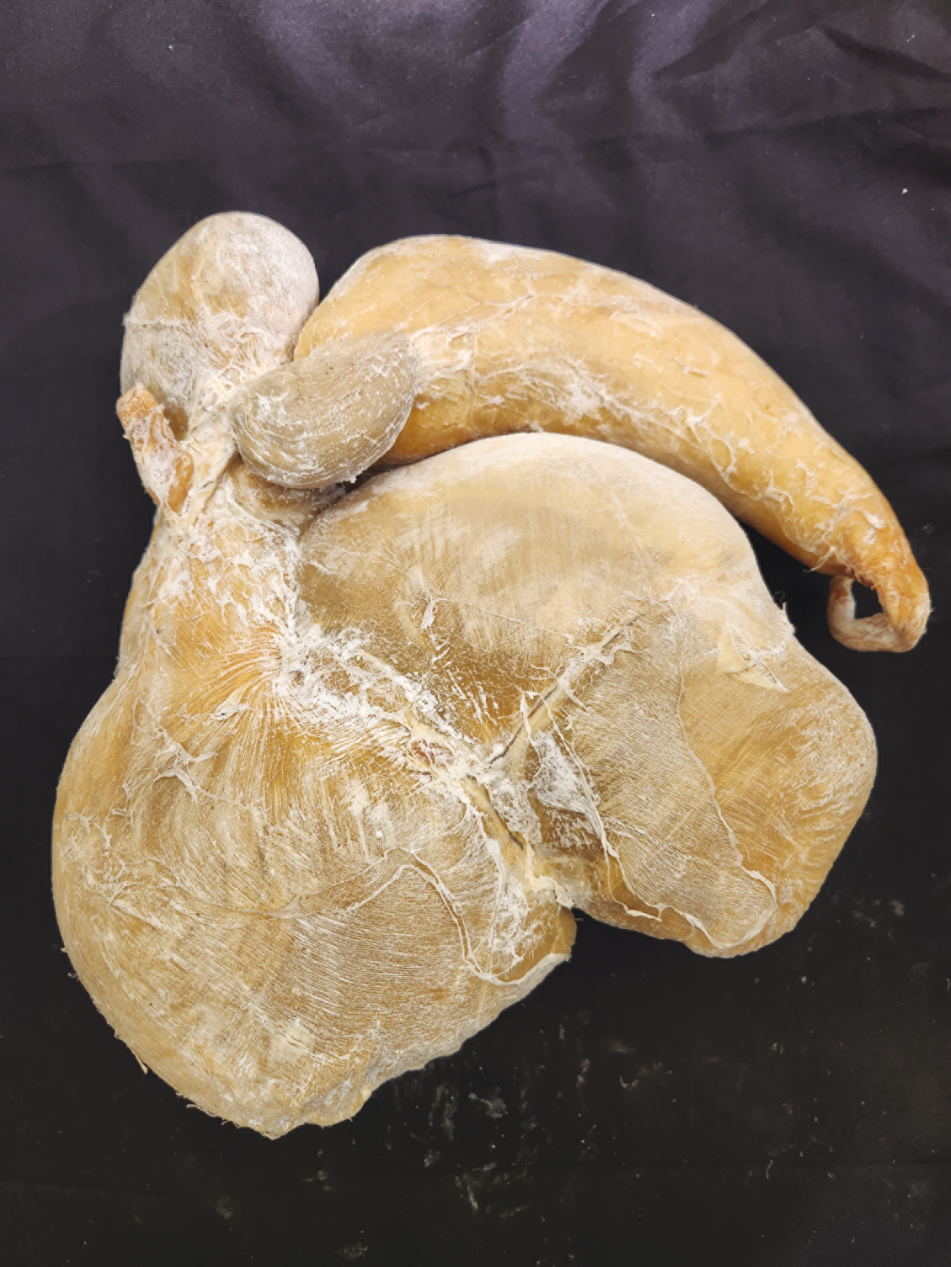
Four-compartment stomach from a goat was preserved using the Elnady Technique (Elnady, 2016) in 2017.

**Figure 16.**
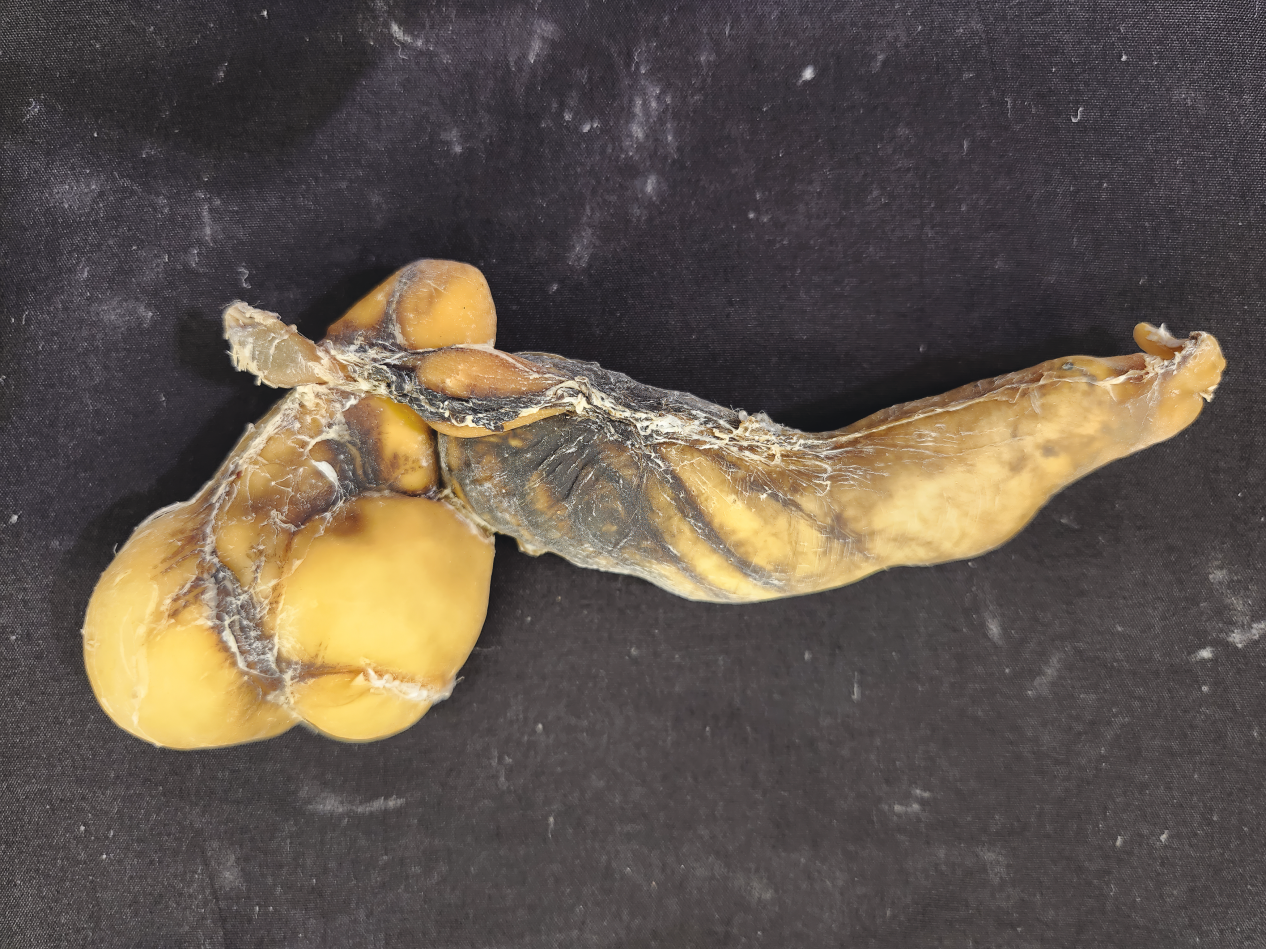
Four-compartment stomachs from a day-old lamb were preserved using the Elnady Technique (Elnady, 2016) in 2019.

Multiple tissue specimens have been preserved using the Elnady Technique (including the female reproductive tract of the cat (Figure 1), goat (Figure 2), horse (Figure 3), pig (Figure 4), and chicken (Figure 5); a digestive tract of cat (Figure 6), chicken (Figure 7), and dog (Figure 8); sheep heart (Figure 9) and kidney (Figure 10); goat rumen (Figure 11), reticulum (Figure 12), omasum (Figure 13), and abomasum (Figure 14); intact four compartment goat stomach (Figure 15); and 1-d old lamb stomach (Figure 16)) at Berry College. The tissue specimens have been used in at least 126 laboratory sections and by at least 1,696 students at Berry College. Some of the specimens have been in use for seven years and are still in usable condition. Others have used the Elnady preservation technique to produce samples for teaching upper respiratory edoscopy in horses (Elnady, 2015), neuroanatomy (Elandy, 2019), and otoscopic evaluation in dogs (Tham et al., 2023) to veterinary students. The Elnady preservation technique has also been used to document and preserve rare cases such as conjoined buffalo calves (Tolba et al., 2020). El-Shafey et al. reported tissue samples were usable with no deterioration 4 yr after preservation using a modified Elnady technique (2022).

Five faculty members responded to the survey. All five faculty members indicated using Elnady Technique preserved tissue specimens with three faculty members having used the specimens four to six times and two faculty members having used the specimens 10 or more times. All five faculty members reported they were extremely likely or very likely to use Elnady Technique preserved tissues in future courses, and all five faculty members also indicated they definitely would recommend or probably would recommend Elnady Technique preserved tissues to other instructors. All five faculty members also strongly agreed or agreed that Elnady Technique preserved tissues are a useful teaching aid. One faculty member commented that smaller specimens “loose shape and color,” and another faculty member said, “the poultry repro tracts are hard to manipulate and show students.” Latex injection immediately after euthanasia and prior to formalin exposure could enhance tissue color (Elnady, 2016; El-Shafey et al., 2022). Bernal et al. reported modification of the Elnady Technique at low temperatures and longer duration along with the use of red vegetable pigment could also enhance the color of the tissue specimens (2022).

In summary, the Elnady Technique provides fully preserved, flexible, and odorless specimens for examination. The specimens may be stored in a dry plastic bag and may be further dissected after preservation. The longevity of the specimens is unknown, but some specimens have been in use for 7 yr. Although there are some challenges with smaller specimens, the Elnady Technique of specimen preservation can produce useful teaching aids for Animal Science instruction.

## Supplementary Material

txae077_suppl_Supplementary_Appendix_S1

txae077_suppl_Supplementary_Appendix_S2
