# Supplementary material for: Evaluation of Elnady preserved tissues as a teaching aid for undergraduate animal science courses
Source: Transl Anim Sci. 2024 May 7;8:txae077. doi: 10.1093/tas/txae077 (PMC11125404; doi:10.1093/tas/txae077)
Supplement: txae077_suppl_Supplementary_Appendix_S1 [file txae077_suppl_supplementary_appendix_s1.docx]

Appendix 1: Email Message

Hello,

You are being invited to participate in a research study to evaluate Elnady preserved tissues as a teaching aid for animal science courses*.* This study is being conducted by Jay Daniel in the Department of Animal Science. You were selected as a possible participant in this study because you may have used Elnady preserved tissue(s) as a teaching aid. There are no costs to you other than time spent for participating in the study. The information you provide will used in a publication on Elnady preserved tissue as a teaching aid for animal science courses. The questionnaire will take about 5 minutes to complete. The information collected may not benefit you directly, but the information learned in this study should provide general benefits to the field of Animal Science.

This survey is anonymous. Do not write your name on the survey. No one will be able to identify you or your answers, and no one will know whether or not you participated in the study. Individuals from the Institutional Review Board may inspect these records. Should the data be published, no individual information will be disclosed.

Your participation in this study is voluntary. By completing the attached survey, printing a copy of your answers, and placing your results in the drop box in A231, you are voluntarily agreeing to participate. You are free to decline to answer any particular question you do not wish to answer for any reason.

If you have any questions about the study, please contact Jay Daniel at (***) ***-****.

The Berry College Institutional Review Board (IRB) has reviewed my request to conduct this project. If you have any questions or concerns about this research project or your rights as a participant, you can contact IRB through Laura Taylor (***) ***-****.
