# Supplementary material for: Evaluation of Elnady preserved tissues as a teaching aid for undergraduate animal science courses
Source: Transl Anim Sci. 2024 May 7;8:txae077. doi: 10.1093/tas/txae077 (PMC11125404; doi:10.1093/tas/txae077)
Supplement: txae077_suppl_Supplementary_Appendix_S2 [file txae077_suppl_supplementary_appendix_s2.docx]

Appendix 2:

Survey:

Question 1.

How many times have you used Elnady preserved tissues as a teaching aid?

- 0-3
- 4-6
- 7-9
- 10 or more

Question 2.

Based on your experience using Elnady preserved tissues as a teaching aid, how likely are you to use Elnady preserved tissues in future courses?

- Extremely likely
- Very likely
- Moderately likely
- Slightly likely
- Not at all likely

Question 3.

Based on your experience using Elnady preserved tissues as a teaching aid, would you recommend Elnady preserved tissues to other instructors?

- Definitely would recommend
- Probably would recommend
- Not sure
- Probably would not recommend
- Definitely would not recommend

Question 4

Overall, Elnady preserved tissues are a useful teaching aid.

- Strongly agree
- Agree
- Neither agree nor disagree
- Disagree
- Strongly disagree

Question 5

If you would like to share any additional comments about Elnady preserved tissues, please feel free to type them below.
